# Interest Forwarding in Named Data Networking Using Reinforcement Learning

**DOI:** 10.3390/s18103354

**Published:** 2018-10-08

**Authors:** Olumide Akinwande

**Affiliations:** Department of Electrical and Electronic Engineering, Imperial College, London SW7 2AZ, UK; oja13@imperial.ac.uk; Tel.: +44-7860847129

**Keywords:** information centric networks, named data networking, cognitive packet networks, random neural networks

## Abstract

In-network caching is one of the key features of information-centric networks (ICN), where forwarding entities in a network are equipped with memory with which they can temporarily store contents and satisfy en route requests. Exploiting in-network caching, therefore, presents the challenge of efficiently coordinating the forwarding of requests with the volatile cache states at the routers. In this paper, we address information-centric networks and consider in-network caching specifically for Named Data Networking (NDN) architectures. Our proposal departs from the forwarding algorithms which primarily use links that have been selected by the routing protocol for probing and forwarding. We propose a novel adaptive forwarding strategy using reinforcement learning with the random neural network (NDNFS-RLRNN), which leverages the routing information and actively seeks new delivery paths in a controlled way. Our simulations show that NDNFS-RLRNN achieves better delivery performance than a strategy that uses fixed paths from the routing layer and a more efficient performance than a strategy that retrieves contents from the nearest caches by flooding requests.

## 1. Introduction

Information-centric networking (ICN) has become one of the leading topics in the next-generation networking and future Internet architecture research. The fundamental concept in ICN is to make content directly addressable, a significant departure from the host-centric communication model of the current Internet. To achieve this, ICN names and addresses data units at the network level, so that in the place of a host address, request packets are routed and satisfied based on the name or identification of the desired content.

This approach is motivated by the increasing domination of Internet traffic by multimedia, effectively making the Internet a content distribution network [1]. Despite the success of the current solutions for efficient information delivery like the content delivery networks (CDNs) and peer-to-peer (P2P) networking, they are limited and complicated by the underlying host-to-host communication [2]. By treating data as fundamental to the architecture, ICN seeks to match the current demands being placed on the Internet and improve on these patch solutions. The separation of content from location enables ICN to support naturally desirable features like in-network caching and multicast communication. In addition to the efficient delivery of content, ICN, using data-centric methods, also addresses significant issues including mobility, security, and quality of service.

Various projects explore the ICN concept, differing mainly in their formats for naming data, caching policies, routing of data objects and requests, and in how they handle mobility and security [3,4]. Of particular interest to us in this work, is the Named Data Networking architecture [5,6] which incorporates a stateful and intelligent forwarding plane in addition to a routing layer. The forwarding plane, also called the *strategy module*, is expected to leverage on both the information provided by the routing layer and the observed packet delivery measurements to make adaptive decisions when forwarding requests. In the general case, the strategy module is defined for each reachable name announced by the routing layer. Furthermore, such an intelligent forwarding plane could relax the stringent convergence and correctness demands commonly placed on the routing layer as explored by the work in [7].

For scalability and practicality reasons, the name-based routing protocol in NDN can only announce and monitor paths leading to the actual publishers and designated repositories, thus leaving the responsibility of exploiting the in-network caching capability, which is vital for delivering the design goals of NDN, entirely to the forwarding strategy. However, most of the proposals for the strategy layer, including the default algorithm adopted by the NDN project, follow a “monitor-the-routing-paths” approach, whereby their forwarding and probing actions are restricted to the interfaces suggested by the routing layer cite [7,8,9,10,11]. This approach limits the forwarding strategy from exploiting local caches closer but outside the routing paths.

In this paper, we develop a dynamic self-aware [12] strategy layer for NDN architectures to offer fast content delivery using local content stores, and we also keep the existing capabilities of the routing layer. The NDN forwarding strategy that we implement exploits the Random Neural Network (RNN) [13] with Reinforcement Learning, similar to the Cognitive Packet Network (CPN) [14,15]. An overlay network with a similar scheme is described in [16].

The rest of the paper is organised as follows. In the remainder of this section we briefly describe the NDN forwarding engine. In Section 2, we review related work on forwarding in NDN, while Section 3 presents a reinforcement learning algorithm for the RNN [13,17]. The *NDN forwarding strategy using reinforcement learning with the RNN* (NDNFS-RLRNN) is detailed in Section 4. In Section 5, we describe the system we evaluate in our simulations, and we discuss our results in Section 6. Finally, conclusions and future work are discussed in Section 7.

### The NDN Forwarding Plane

NDN uses two types of packets for communication. Requests are issued using *Interest packets*, while *Data packets* are used to carry the requested contents. Both packets include the name or identification of the desired data object. An Interest is uniquely identified by its name and nonce values, where the nonce is randomly generated by the requesting application. In addition, the Data packet also includes a digital signature, created by the content producer, that binds the name with the content.

An NDN content router (CR) maintains three data structures for implementing the forwarding plane: a *content store* (CS), a *pending interest table* (PIT) and a *forwarding information base* (FIB). The CS acts as a temporary cache for Data. It is used to implement in-network caching and can satisfy content requests. A CR caches traversing Data packets according to a well-defined caching policy. The PIT is used to keep track of the unanswered Interests. The PIT also aggregates information about subsequent Interests that arrive and match any of its entries; the CR then decides whether or not to forward such Interests. The information recorded in a PIT entry will include the content name, a list of arrival and outgoing links, and the arrival and forwarding times of the Interests. Since Interests can be uniquely identified, the PIT can detect when an Interest loops. Moreover, by keeping the forwarding state in the PIT, delivery performance can be estimated and used by the strategy module in influencing future forwarding decisions. Finally, the FIB contains a mapping of the reachable content names to the outgoing interfaces of the CR. The FIB is populated and updated by a name-based routing protocol. The strategy module uses the routing information in the FIB in forwarding Interests.

It is important to mention that the format for naming data objects is an active area of research in ICN [3,18]. NDN adopts a hierarchical structure in naming content which is similar to URLs. When a CR receives an Interest, it first checks its CS for matching data objects. According to the blueprint for the NDN architecture in [8], Data in the CS is said to match an Interest if the content name of the Interest is a prefix of the content name of the Data. NDN also allows the requesting application to have some control over which Data packets are satisfactory through an optional *selector* field in the Interest packet. In the event of a match, the Data packet is returned on the arrival interface of the Interest packet. Otherwise, a lookup, which should also consider the entries in the selector field, is performed in the PIT for an exact match. A match in the PIT means the CR is already expecting a response that will satisfy the received Interest, so the corresponding PIT entry is updated and the strategy module decides whether or not to forward the Interest packet. Otherwise, the longest-prefix match entry in the FIB is found, and the strategy module decides the outgoing interface(s) to forward the Interest. If there is no match in FIB, the CR has no knowledge of the desired content, so the Interest is discarded.

Data packets, on the other hand, are forwarded along the reverse path used by their corresponding Interest packets by using the information recorded in the PITs. On receiving a Data packet, the CR performs a lookup in the PIT for a matching entry. If there is a match, the CR sends a copy of the Data packet on all the interfaces listed under “incoming” in the entry. The CR then deletes this entry from the PIT and decides whether or not to cache the Data packet. Otherwise, the content is considered unsolicited, and the Data packet could be discarded without caching.

## 2. Related Work

It is shown in [19] that coupling caching and forwarding is essential in ICN to significantly benefit from ubiquitous caching. The authors first studied an optimal policy, the *ideal Nearest Replica Routing* (iNRR), in which CRs forward their requests to the nearest possible replica with the help of an “oracle” that keeps track of the network’s caching state. Then, due to the cost of realising such an oracle, they proposed practical implementations where CRs periodically explore a given neighborhood in the network by flooding the request. A review of caching strategies that have been evaluated in the ICN research is presented in [20].

In the NDN context, Jacobson et al. [8] proposed forwarding an Interest along all the interfaces suggested by the routing layer excluding the interface the Interest arrives on, referred to as the *multicast strategy*. The *best route strategy* [9] forwards interests using the available upstream with the lowest routing costs. The Adaptive SRTT-based Forwarding Strategy (ASF) is proposed in [7]. ASF sends an Interest using the upstream with the lowest measured SRTT among those put forward by the routing protocol. To gather measurements, ASF also probes alternative interfaces at intervals. The current NDN forwarding strategy [6,10,21] combines interface ranking and colour classification to decide where to forward Interests. Interfaces suggested by the routing protocol are first classified using a colour scheme according to how well they are known to return Data, then the interfaces are ranked in each class using some metric, usually the smoothed round trip time (SRTT). The forwarding logic is to use the highest ranked available interface in the best possible classification. NDN also introduced Interest to address the inefficiencies that result from the dangling states in the PIT caused by unsatisfied Interests. When it cannot forward nor satisfy an Interest, a CR responds with an Interest NACK; the Interest NACK also carries a code describing the reasons. Therefore, Interest NACKs can help the network to quickly and informedly detect faults and, when possible, try other forwarding options.

To eliminate undetected loops in NDN, the *Strategy for Interest Forwarding and Aggregation with Hop-Counts* (SIFAH) is proposed in [11]. In SIFAH, a CR accepts to forward an Interest only if there exists, based on distance information to the content source, a neighbour node that moves the packet closer to the content.Otherwise, an Interest NACK is returned. The distance information used in SIFAH is the number of hops to the content repository, and it is provided to the forwarding plane by the routing protocol. Also, by replacing nonce values with distance information, SIFAH reduces the memory overhead incurred by the PIT. While SIFAH guarantees a correct forwarding strategy, it achieves this by limiting the dynamism of the forwarding plane in making adaptive decisions. In addition, the conditions it uses for loop detection, and therefore for forwarding Interests, are sufficient conditions. This means that an interface can fail these conditions and yet not lead to an Interest loop being formed. Hence, the possibility of unnecessarily denying service to Interests exists.

Another class of forwarding strategies referred to as the *multipath* strategies [22,23,24], dynamically assign a forwarding weight or probability to each interface of a CR, which determines the proportion of Interest traffic sent on it. The main aim here is to achieve load balancing and manage congestion in the network.

In our work, we propose implementing the strategy module of the NDN architecture using an online learning algorithm. Reinforcement learning has been previously proposed for the NDN forwarding strategy. In [25] the *multi-armed bandits strategy* (MABS) is developed which assumes no knowledge of path information from the routing layer. As a result, when no forwarding information is available, the CR floods a request on all its interfaces. *INFORM*, presented in [26] and inspired by the Q-routing algorithm [27], adopts a more similar approach to ours by leveraging on the routing layer. INFORM alternates between exploration and exploitation phases when making forwarding decisions. In the initial exploration phase when no learning has occurred, a received Interest is sent using the best interface according to the information in the FIB, and a copy of the Interest is also sent on a randomly selected interface. The same actions are repeated in subsequent exploration phases except that the best interface will be the one learnt by the algorithm. Only the best interface computed after an exploration phase is used during the subsequent exploitation phase. In these methods, the authors do not address the handling of Interest NACKs, which becomes significant because the exploration of the network, necessary for the learning algorithm, increases the possibilities of Interest loops.

Our approach is inspired from the CPN routing protocol which, in most of its implementations, employs a reinforcement learning algorithm using the RNN [13] to establish and maintain connections between network nodes. The algorithm used has been shown to possess fast convergence properties during an initial learning phase and good sensitivity to environmental changes [17], hence its success in CPN routing. An early review of the variations, applications, and performance evaluations of the CPN can be found in [28].

## 3. Reinforcement Learning and the Random Neural Network

The RNN is a neural network model inspired by queueing networks [29] where neurons send and receive both positive and negative signals. The positive signals are called *excitatory*, while the negative signals are called *inhibitory*. It has been used in many applications, including image and video compression [30,31], the recognition of tumours from Magnetic Resonance Images of the human brain [32], toxicity prediction of chemical compounds [33], web search [34], and network routing and cloud management [35,36,37,38,39]. It is a special instance of the family of stochastic networks known as G-Networks [40,41,42,43,44,45] which have many different areas of application.

Table 1 lists the notation for the RNN model. The state of a neuron *i* in the network at any time is represented by a non-negative potential value $k_{i}\left(t\right)$, so that the vector:

$$k\left(t\right)=(k_{1}\left(t\right),k_{2}\left(t\right),\ldots ,k_{n}\left(t\right))$$

describes the state of an RNN at any time *t*. An incoming signal either increases a neuron’s potential by one if it is excitatory or decreases it by the same amount if it is inhibitory. An excited neuron *i*, that is, one with non-zero potential ($k_{i}\left(t\right)>0$), fires randomly by sending out excitatory or inhibitory signals to other neurons or to the outside of the network. Whenever a neuron fires, its potential reduces by one. Signals can also arrive at a neuron from outside the RNN.

For each neuron *i* in an *n*-neuron network:

(1) $$\sum_{j}^{n}\left[p^{+}\left(i,j\right)+p^{-}\left(i,j\right)\right]+d\left(i\right)=1.$$

It was shown in [46] that the RNN with exponential firing intervals and Poisson exogenous signal arrivals has the product form solution:

(2) $$p\left(k\right)=\prod_{i=1}^{n}\left(1-q_{i}\right)q_{i}^{k_{i}},\;q_{i}=\frac{\lambda_{i}^{+}}{\left(r_{i}+\lambda_{i}^{-}\right)},$$

where $p\left(k\right)=\lim_{t\to \infty }Pr\left[k\left(t\right)=k\right]$, $k=(k_{1},k_{2},\ldots ,k_{n})$, is the network’s stationary probability distribution; $q_{i}$ is the steady state probability that neuron *i* is excited. $\lambda_{i}^{+}$ and $\lambda_{i}^{-}$ represent the overall flow of positive and negative signals, respectively, at neuron *i*. For i=1,2,…,n, $\lambda_{i}^{+}$ and $\lambda_{i}^{-}$ satisfy the following system of non-linear equations whose solution exists and is unique [47]:

(3) $$\lambda_{i}^{+}=\sum_{j}\left[q_{j}r_{j}p^{+}\left(j,i\right)\right]+\Lambda_{i}$$

(4) $$\lambda_{i}^{-}=\sum_{j}\left[q_{j}r_{j}p^{-}\left(j,i\right)\right]+\lambda_{i}$$

For adaptive routing applications, the recurrent RNN model is normally used. This RNN is fully connected and has as many neurons as there are possible outgoing links at the node it resides, that is, a neuron represents a possible forwarding decision. The weight of each RNN connection (i,j) is the emission rate of positive or negative signals from neuron *i* to neuron *j*. That is,

$$w^{+}\left(i,j\right)=r_{i}p^{+}\left(i,j\right),\;w^{-}\left(i,j\right)=r_{i}p^{-}\left(i,j\right)$$

Introducing the above expressions and assuming signals do not leave the network (d(i)=0), we can rewrite Equation (1) as:

(5) $$r_{i}=\sum_{j=1}^{n}\left[w^{+}\left(i,j\right)+w^{-}\left(i,j\right)\right]\;i=1,2,\ldots ,n$$

The matrices of the weights of the connections: $W^{+}=\left\{w^{+}\left(i,j\right)\right\},\;W^{-}=\left\{w^{-}\left(i,j\right)\right\},$ are important parameters whose values are continuously modified during the learning process.

The RNN is initialised with all the weights equal. This is done by choosing a firing rate $r_{i}$ and using Equation (5) to solve for the initial weight value. That is, $w=\frac{r_{i}}{2(n-1)},$ where *w* is the initial value of all weights; remember that $w^{+}\left(i,i\right)=w^{-}\left(i,i\right)=0\;\forall i$.

The pseudocode in Algorithm 1 illustrates how reinforcement learning can be implemented using the RNN. The reinforcement learning algorithm used is inspired from the *E-rule* scheme in [17], and it assumes that a reward value can be estimated for every decision. In this approach, the outcome of a previous decision, $R_{l}$, is compared with an internal expectation, $T_{l-1}$, of the neural network and the RNN receives reinforcement based on the result of this comparison. The reinforcement can either be *positive* if the outcome is considered a success, or *negative* otherwise. In the illustration in Algorithm 1, the *l*-th decision is considered to be a success if $R_{l}\geq T_{l-1}$; otherwise it is a failure. A success leads to a significant increase in the excitatory weights going into the corresponding neuron and a small increase in the inhibitory weights leading to the other neurons. Otherwise, the inhibitory weights of the neuron assigned to the decision is significantly increased and the excitatory weights into the remaining neurons are slightly increased.

**Algorithm 1** The RNN training process**INPUT:**$T_{l-1}$, $W^{+}$, $W^{-}$, internal expectation and weight matrices before the *l*-th reward is received; 0<α<1. **INPUT:**${R^{i}}_{l}$, the *l*-th received reward obtained when the *l*th decision was to select output link *i*. **OUTPUT:**$q_{i}$, ∀i=1,2,...,n, the excitation probabilities for an RNN with *n* neurons    **if**
${R^{i}}_{l}\geq T_{l-1}$
**then**
     /* *l*-th decision is a success*/      **for** each neuron *j* in the RNN, j≠i
**do**
         $w^{+}\left(j,i\right)\leftarrow w^{+}\left(j,i\right)+{R^{i}}_{l}$         **for** each neuron *k* in the RNN, k≠j
**do**            $w^{-}\left(k,j\right)\leftarrow w^{-}\left(k,j\right)+\frac{{R^{i}}_{l}}{n-2}$   **else**     /* *l*-th decision is not a success*/     **for** each neuron *j* in the RNN, j≠i
**do**         $w^{-}\left(j,i\right)\leftarrow w^{-}\left(j,i\right)+{R^{i}}_{l}$         **for** each neuron *k* in the RNN, k≠j
**do**            $w^{+}\left(k,j\right)\leftarrow w^{+}\left(k,j\right)+\frac{{R^{i}}_{l}}{n-2}$   /* Normalizing the weights */   **for** each neuron *j* in the RNN **do**     $r_{j}^{*}\leftarrow \sum_{m}^{n}\left[w^{+}\left(j,m\right)+w^{-}\left(j,m\right)\right]$     **for** each neuron *k* in the RNN, k≠j
**do**         $w^{+}\left(j,k\right)\leftarrow w^{+}\left(j,k\right)*\frac{r_{j}}{r_{j}^{*}}$         $w^{-}\left(j,k\right)\leftarrow w^{-}\left(j,k\right)*\frac{r_{j}}{r_{j}^{*}}$   /*solve the *n* non-linear simultaneous equations in (2) for each $0\leq q_{j}\leq 1$ */   /* We use a fixed-point iteration, starting with $q_{j}^{0}=0.5$∀j=1,2,...,n */      $q_{j}^{k+1}\leftarrow min\left[1,\frac{\sum_{m}\left[q_{m}^{k}w^{+}\left(m,j\right)\right]+\Lambda_{j}}{r_{j}+\sum_{m}\left[q_{m}^{k}w^{-}\left(m,j\right)\right]+\lambda_{j}}\right]$   /* Updating the internal expectation */   $T_{l}\leftarrow \alpha T_{l-1}+\left(1-\alpha \right)R_{l}$ /* α is to be chosen closer to 1 */

After updating the weights, Algorithm 1 also shows a renormalisation of the weights. This is to prevent ever-increasing weights, and the fact that it is the relative magnitudes of the weights that determine the state of the network justifies this step. The new firing rate $r_{i}^{*}$ for each neuron is first evaluated using the updated weights in Equation (5). Then, for each neuron *i* the normalised weights are computed as $w^{+}\left(i,j\right)=w^{+}\left(i,j\right)*\frac{r_{i}}{r_{i}^{*}},w^{-}\left(i,j\right)=w^{-}\left(i,j\right)*\frac{r_{i}}{r_{i}^{*}},\;j=1,2,\ldots ,n,$ where $r_{i}$ is the initial or reference firing rate.

Finally, the excitation probabilities $q_{i}$ for all the neurons are evaluated by solving the non-linear system of equations $q_{i}=\frac{\lambda_{i}^{+}}{\left(r_{i}+\lambda_{i}^{-}\right)},\lambda_{i}^{+}=\sum_{j}\left[q_{j}r_{j}p^{+}\left(j,i\right)\right]+\Lambda_{i},\lambda_{i}^{-}=\sum_{j}\left[q_{j}r_{j}p^{-}\left(j,i\right)\right]+\lambda_{i}.$

The common approach to solving these equations is using the simple fixed-point iteration as shown in Algorithm 1. At any given time, the neuron with the highest excitation probability *q* is considered the best decision.

Finally, the internal expection is updated with the new reward using $T_{l}=\alpha T_{l-1}+\left(1-\alpha \right)R_{l},$ where 0<α<1.

## 4. NDN Forwarding with Reinforcement Learning Using the RNN

We present NDNFS-RLRNN that uses reinforcement learning (RL) with the RNN for the strategy module per prefix in the NDN architecture, which operates with a form of smart Opportunistic Communication [48], supported by a name-based routing protocol with online measurements from the state recorded in the PIT. Our goal is to exploit the convergence properties of the RL with RNN algorithm which have been well studied in [17,49] algorithm to effectively search for local content store hits.

In NDNFS-RLRNN, an RNN is created for a prefix in the FIB and its creation is triggered by a new Interest for the corresponding prefix. In its initial state, the RNN only knows of the routing preferences for its prefix and is yet to be updated by packet delivery measurements. Here, NDNFS-RLRNN’s forwarding decision will be to use the best interface according to the routing layer. Each RNN per prefix at the CR has as many neurons as the number *k* of outgoing links of the router, and the RNN has $k^{2}$ weights, and if the router has $n_{D}$ active destinations, the router will have a total of $n_{D}$ RNNs to handle each of the destinations, or a total of $n_{D}\times k^{2}$ entries. A conventional routing table at the NDN router will have $n_{S}\times n_{D}\times k$ entries for $n_{S}$ active sources. Thus when $n_{S}>k$, our scheme uses a smaller data structure per router than a conventional NDN scheme [50].

On the arrival of a Data packet, a reward value is computed for the arrival interface and used to update the RNN as illustrated in Algorithm 1. The goal of our distributed reinforcement learning is to minimise the delay for retrieving contents, so we estimate reward using the RTT values. Let ${T^{j}}_{I}$ be the time an NDN router forwards an Interest along an interface *j* and ${T^{j}}_{D}$ be the arrival time of the Data packet satisfying the Interest which also arrives on the same interface, we can estimate the reward, $R^{j}$ as the inverse of the RTT using

(6) $$R^{j}={\left({T^{j}}_{D}-{T^{j}}_{I}\right)}^{-1}$$

**Figure 1:**
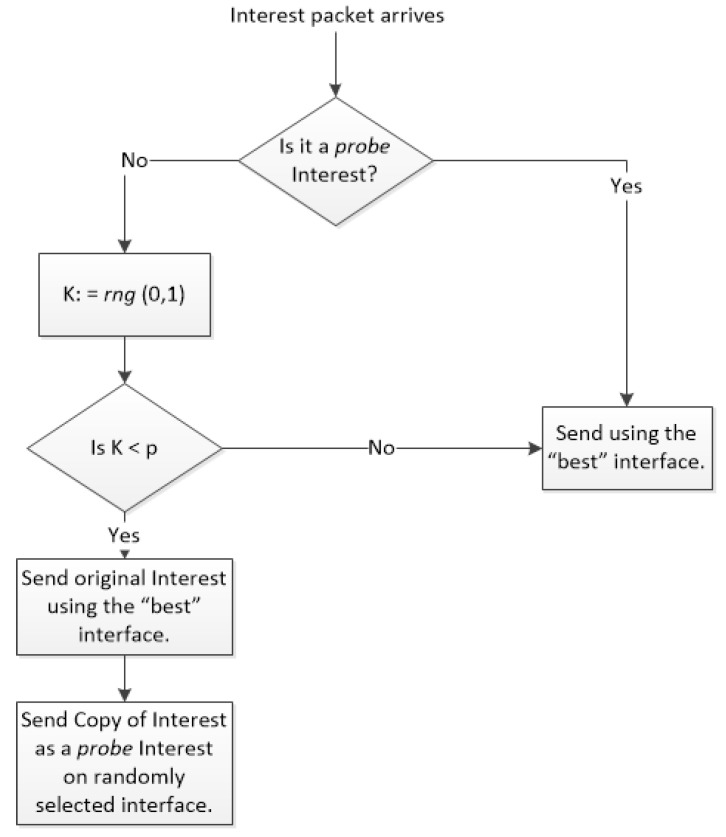
Interest forwarding and probing process of NDNFS-RLRNN. It uses the ϵ-greedy approach for the probing. *rng*(0,1) is a random number generator that returns a number in the range [0,1). *p* is the probe parameter of the RNN. It is assumed that the RNN is no longer in its initial state and that forwarding is possible so that the best interface refers to the interface corresponding to the most excited neuron of the RNN.

For an updated RNN, the forwarding decision of NDNFS-RLRNN is, excluding the arrival interface, with a high probability *p* only the interface corresponding to the most excited neuron is used or with probability (1−p) a probing decision is made. We refer to *p* as the *probe parameter*. Before explaining the probing process, we first introduce the idea of marking an Interest packet for probing. This means an Interest can be identified at the CRs as either a “normal" Interest or a probe Interest. The probing process involves sending two packets: the original Interest which is sent along the best known interface and a copy which is sent as a probe Interest on a randomly selected interface. We impose that a CR only forwards a probe Interest along the best known interface. The motivation for this probing process is to control exploration in order to reduce the possibility of unnecessarily using longer paths to retrieve contents and to manage overhead. In Figure 1 we use a simple flowchart to summarise the Interest forwarding and probing process of NDNFS-RLRNN.

We have also adopted the use of Interest NACKS as in the current NDN in our approach. When a CR can neither satisfy nor forward an Interest, it responds using an Interest NACK. Clearly, the reward estimation is explicit when an Interest is satisfied. However, this is not the case when an Interest NACK is returned or when no response is received before the Interest times out. In such cases, the reward can be estimated as:

(7) $$R^{j}={\left[c\left(T-{T^{j}}_{I}\right)\right]}^{-1},$$

where *T* is the current time and *c* is a constant large enough such that a negative reinforcement is applied. Therefore, before deleting a PIT entry, all the interfaces that failed to return data will receive negative reinforcement based on the reward computed in Equation (7). On the arrival of an Interest NACK in response to a request, the CR will first try to resend the Interest using the routing layer preferences. If retransmission is impossible, an Interest NACK is returned on all the expecting interfaces, and the RNN for this prefix returns to the initial state.

To reduce the inefficiencies that could be introduced by out-of-date information and for efficient use of resources, when an RNN receives no feedback for $T_{r}$ time units, it is deleted. As a result, an RNN will remain in the system only if it is considered active according to the above $T_{r}$ condition. This is necessary to manage the limited resources at each CR. Furthermore, a limit could be set for the number of RNNs at a node depending on the available network resources, such that new Interests that arrive after this limit is reached will be sent using the routing layer options without any attempt for a local search.

## 5. System and Scenario Description

While there is no complete consensus on the evaluation of information-centric networks, the scenarios we consider in our simulations are in line with much of the related literature, especially with respect to the workload and the network topology used. In the remainder of this section, we describe the models and the assumptions adopted for the system we analyze.

### 5.1. Request Arrival Model

The most common approach in analysing caching system is the assumption of the independent reference model (IRM) [51] to model the arrival of requests, with a Zipf-like distribution to represent the content popularity. IRM describes the requests arriving at a cache for a fixed catalog of items as forming a sequence of independent, identically distributed random variables. In other words, the probability that a request is for an item is a constant and independent of the past requests.

The IRM has the advantage that it is simple and has enabled the development of tractable models in the analysis of caching systems [52,53,54]. Although IRM does not adequately capture phenomena like *temporal locality* and *spatial locality* which have been shown to exist in real traces [55,56], other studies [57,58] argue that their long-term effect can be well accounted for by careful choice of a Zipf-like distribution to represent content popularity. Temporal locality occurs when recently accessed items are more likely to be requested in the near future while spacial locality describes the situation where the request for an item becomes more likely because a “similar" item was referenced in the recent past.

### 5.2. Content Popularity

The choice of the popularity distribution is an important factor that determines the performance of in-network caching in ICN. The Zipf’s law is the favoured choice to model popularity in ICN [19,59,60].

Zipf’s law, popularized initially in Statistical Linguistics, predicts, for a catalog of *N* items, that the probability of referencing the *i*-th most popular item is:

(8) $$\rho_{N}\left(i\right)=\frac{\frac{1}{i^{\alpha }}}{\sum_{i=1}^{N}\frac{1}{i^{\alpha }}},$$

where the *skew factor*, α, is a constant characterising the distribution.

The more general Mandelbrot-Zipf (Mzipf) distribution is also adopted in some analysis [61,62]. In MZipf,

(9) $$\rho_{N}\left(i\right)=\frac{\frac{1}{{\left(i+q\right)}^{\alpha }}}{\sum_{i=1}^{N}\frac{1}{{\left(i+q\right)}^{\alpha }}},$$

where α and *q* both characterise the distribution. Equation (9) reduces to (8) when q=0.

There is also no consensus on the parameter settings for either distributions. However, the values used in the literature are informed by those arrived at from large-scale studies of actual traces observed at ISPs and CDNs [57,63,64,65]. As a result, values of α∈[0.6,2.5] and q∈[0,50] are common, while [24,66,67] include uniform popularity (α=0) in their evaluations.

In our tests, we adopt the MZipf distribution to represent content popularity, and we use values within these ranges which are realistic, considering the catalogue sizes we consider, to characterise the distribution.

### 5.3. Network Topology

With respect to the network segment used in our tests, we have also followed the trend in recent ICN studies by adopting a real ISP topology. Furthermore, we assume that the nodes and links within the topology have identical resources in terms of cache allocation and link capacity. Finally, we assume in all the tests carried out that the system operates below congestion.

## 6. Results and Discussion

In this section, we present initial evaluations of the performance of NDNFS-RLRNN through extensive simulations using the *ndnSim* [68], an NS-3-based simulator which already exists for NDN.

**Figure 2:**
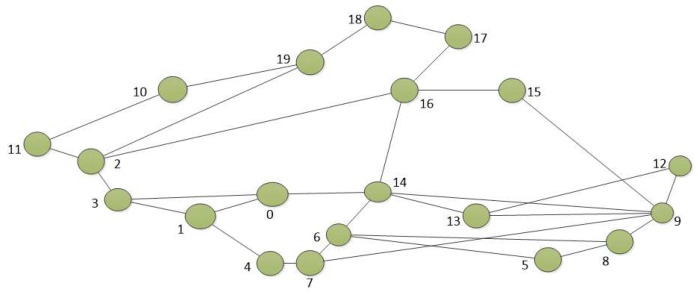
*Elibackbone* topology. The network consists of 20 nodes and 30 links.

For the simulations, we consider an NDN-based network connecting users to content servers. We use the real topology *Elibackbone* shown in Figure 2 according to the dataset in [69]. We consider a single access router for the network through which requests are served from the content servers. At each node, except the access node, external request arrivals are Poisson process with a rate 5 request packets per second, and each data object is 10 KB in size. All the simulations begin with empty caches at the nodes.

Furthermore, we compare NDNFS-RLRNN with the *Adaptive SRTT-based Forwarding Strategy* (ASF) [7], and a Nearest Replica Routing (NRR) strategy [19]. The *ndnSim* has the ASF algorithm pre-installed. The ASF strategy uses the upstream with the lowest measured SRTT and probes alternative interfaces suggested in the FIB at intervals. The length of the probing interval is reduced from the default value of 60 s to 3 s to increase probing, and we install all possible routes in the FIB such that no loop exists in the forwarding. For the NRR strategy, we implement a multicast algorithm that sends each request on all the interfaces of a node except the arrival interface, which guarantees that the requested data objects are retrieved from the closest caches. Other approaches are possible where the flooding is periodic as suggested in [19], but we have chosen this simple method that avoids parameter settings. The forwarding strategies are implemented per content in the catalogue.

Since the cache states in the network will influence the performance, the forwarding strategies are compared under three different caching policies from the literature:

- Leave copy everywhere combined with LRU replacement policy (*LCE*): Here, the CRs cache every data packet received.
- Betweenness centrality policy and LRU (*Betw*): In *Betw*, proposed in [70], only the routers with the highest betweenness centrality values along the delivery path cache the content. These values are computed offline using the betweenness centrality definition in graph theory.
- *ProbCache and LRU*: Here the decision to cache a content at a CR is based on a defined probability. We set this probability using the proposed *ProbCache* Algorithm [71], which computes the caching probability when a content arrives at a node by weighing the cache capacity along the remainder of the delivery path with the relative position of the router along this path, as shown in Equation (10).
  (10) $$p\left(x\right)=\frac{\sum_{i=1}^{c-x+1}N_{i}}{kN}$$
  where *c* is the total number of content routers on the path from requester to content source, *x* is the position of the current node along this path, $N_{i}$ is the cache capacity at node *i*, *N* is the average cache capacity along the full path, and *k* is a constant denoting a target cache capacity along a path.

LCE and *ProbCache* are ubiquitous caching policies differentiated by their cache eviction rates. By ubiquitous, we mean that nodes in the network are not prioritised in any way when caching. *Betw*, on the other hand, tries to cache predominantly at the most “central" nodes in the network. These policies represent three categories of on-path caching strategies in the literature.

To evaluate the performance of the forwarding strategies, we measure and report the *cache hit rate* and the *network load per request*. The cache hit rate is measured as the proportion of the requests arriving into the network which are satisfied from the local caches. On the other hand, the network load or overhead is the total number of hops traversed by the network packets, which includes Interests, Data packets and Interest NACKs, per request sent into the network.

Finally, we run each simulation scenario for 400 s, and all metrics are obtained through the mean and standard deviation of 20 randomised simulation runs.

### 6.1. Probe Parameter

We investigate the impact of the probe parameter (*p*) of the NDNFS-RLRNN algorithm. This parameter determines how often alternative paths are explored by a CR. In this simulations, we consider a uniform content popularity model across the network with Mzipf parameters set at α=1.0 and q=5.0. By uniform popularity, we mean that request arrivals at each CR in the network are characterized by the same MZipf parameters. We fix the catalogue size at 1000 data objects, and each CR is equipped with a cache size of 1% of the catalogue.

**Figure 3:**
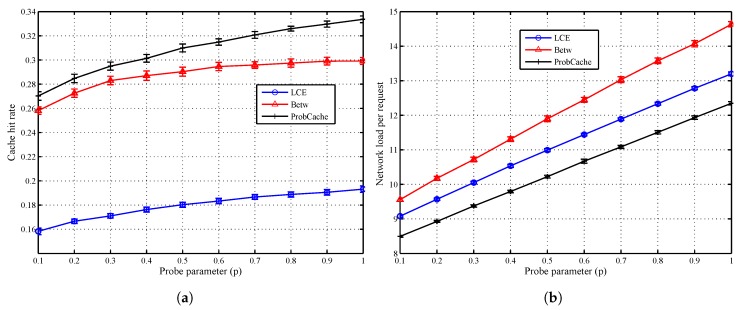
Evaluation of the probe parameter (p) under different on-path caching policies. (**a**) Cache hit rate; (**b**) Network load per request. Each CR is equipped with a cach size of 1% of the content catalogue. Content popularity is modelled according to the Mandelbrot-Zipf (MZipf) distribution with the settings: α=1.0 and p=5.0. The results reported are the average of 20 randomized simulation runs.

In Figure 3, we observe that for uniform content popularity in the network, increasing the exploration of the network, as a result of increasing *p*, increases the cache hit rates at the cost of increased network overload. In general, the *ProbCache* policy produces the best cache hit performance because it combines better redundancy and eviction properties compared with the other policies. The LCE produces the worst performance because of its characteristic high redundancy and high eviction rate. Although *Betw* reduces redundancy compared with LCE, there is a high rate of eviction at the most “central” nodes.

Interestingly, while we observe a steadier increase in the hit rates as *p* increases for both the LCE and *ProbCache* policies, for *Betw*, the benefit reduces as *p* is increased. In fact, 90% of the improvement from increasing *p* from 0.1 to 1.0 is already attained at p=0.6 for Betw compared with about 70% for both LCE and *ProbCache*. Since *Betw* caches contents mostly at specific nodes, NDNFS-RLRNN can search these locations considerably well enough such that further increasing exploration becomes less cost-effective. As expected, Figure 3b shows the overhead cost of increasing the probe parameter.

### 6.2. Catalogue Size

**Figure 4:**
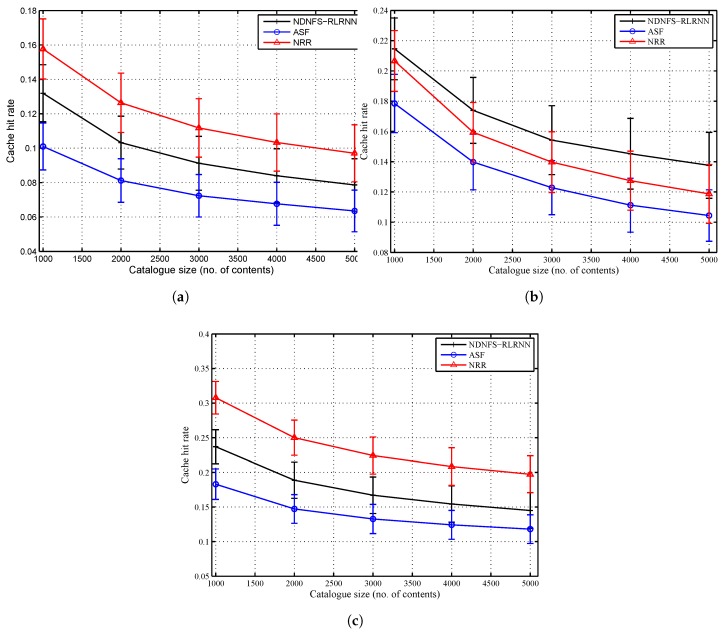
Cache hit rate performance of NDNFS-RLRNN (p=0.3), ASF and NRR for different cache size to catalogue size ratios, under: (**a**) LCE caching policy, (**b**) *Betw* caching policy and (**c**) *ProbCache* caching policy. The cache size of each CR is fixed at 10 data objects while we vary the catalogue size fom 1000 to 5000 data objects. Content popularity is modelled according to the Mandelbrot-Zipf (MZipf) distribution with the settings: p=5.0,α∈[0.6,1.2]. The results reported are the average of 20 randomized simulation runs.

**Figure 5:**
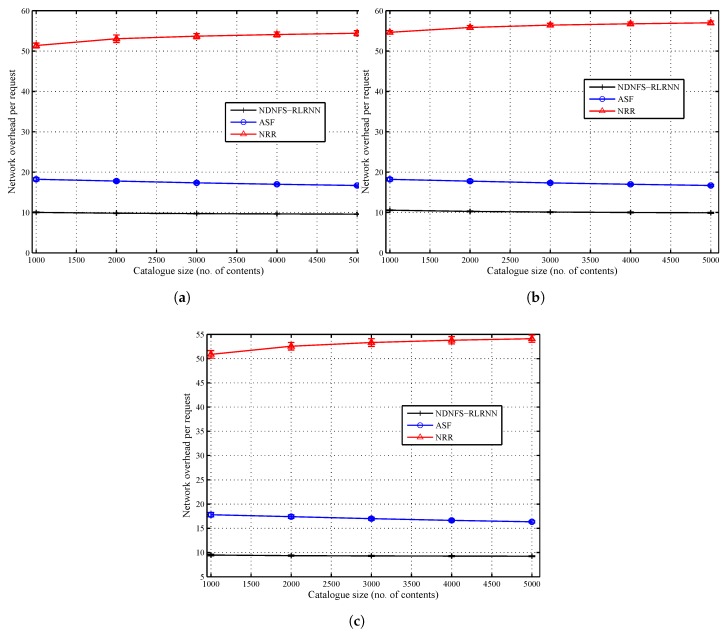
Network load performance of NDNFS-RLRNN (p=0.3), ASF and NRR for different cache size to catalogue size ratios, under: (**a**) LCE caching policy, (**b**) *Betw* caching policy and (**c**) *ProbCache* caching policy. The cache size of each CR is fixed at 10 data objects while we vary the catalogue size from 1000 to 5000 data objects. The cache size of each CR is fixed at 10 data objects. Content popularity is modelled according to the Mandelbrot-Zipf (MZipf) distribution with the settings: p=5.0,α∈[0.6,1.2]. The results reported are the average of 20 randomized simulation runs.

In Figure 4 and Figure 5, we compare the three forwarding strategies and show the impact of the cache size to the catalogue size ratio. In general, performance diminishes as this ratio reduces. We observe that with low exploration, NDNFS-RLRNN can exploit in-network caching better than ASF for all the considered scenarios. With respect to the cache hit rate, it provides a performance improvement compared with ASF of between 23–30%, 20–32%, and 22–30% under the LCE policy, *Betw* policy, and *ProbCache* policy, respectively. Also, NDNFS-RLRNN provides an improvement under the *Betw* policy compared with the NRR strategy of between 3–16%. It achieves these improvements with less overhead as shown in Figure 5c; and as expected, the overhead NRR incurs is significantly higher than the other two forwarding strategies.

The network overhead result as shown in Figure 5 is a measure of the cost to network incurred by the forwarding strategies. The probing of interfaces introduces additional costs because it increases the number of Interests processed in the network and, as a result, the number of Data packets and Interest NACKs. As a result, NRR incurs significantly higher overhead compared with the other forwarding strategies because it tries to flood the network with every request. The ASF is similar, in terms of probing, to the NRR except that the probing is periodical and restricted to a subset of the interfaces at each CR. This explains why NRR generates more than 2.5 times the overhead for ASF in all the considered scenarios. Moreover, NDNFS-RLRNN incurs the least overhead among the considered forwarding strategies primarily because of how it tries to take advantage of the probing process. Also, the low probing parameter value of 0.3 and the use of *probe* Interests to control the network exploration are contributing factors.

Finally, the relatively high deviation from the mean values, compared to the other results, can be explained by the randomization of the content distribution at the CRs.

### 6.3. Content Popularity

**Figure 6:**
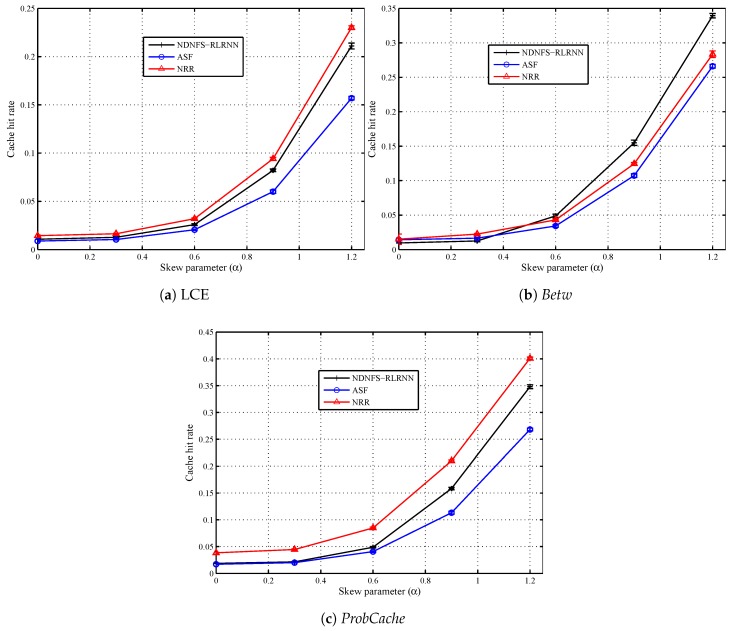
Cache hit rate performance of NDNFS-RLRNN (p=0.3), ASF and NRR for different uniform content popularity distributions, under: (**a**) LCE caching policy, (**b**) *Betw* caching policy and (**c**) *ProbCache* caching policy. Content popularity is modelled according to the Mandelbrot-Zipf (MZipf) distribution with the settings: p=5.0,α=0.0,0.3,0.6,0.91.2. Each CR is equipped with a cache size of 0.4% of the content catalogue. The results reported are the average of 20 randomized simulation runs.

**Figure 7:**
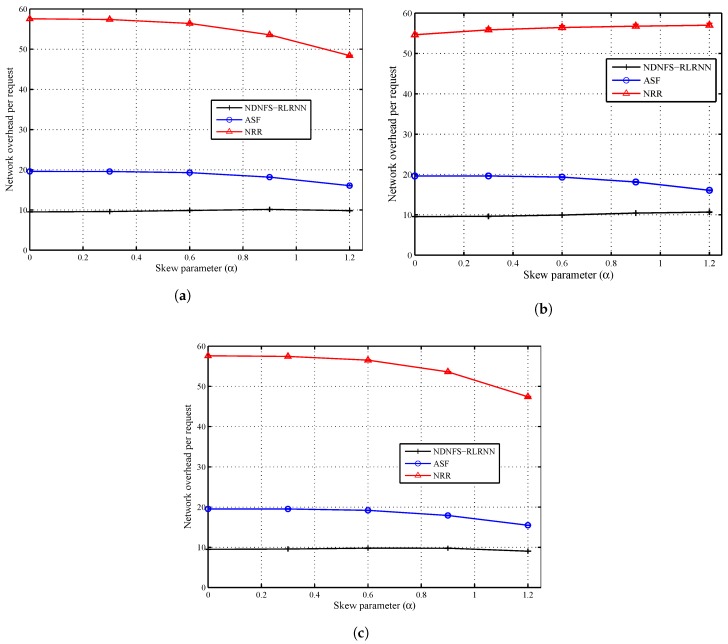
Network load performance of NDNFS-RLRNN (p=0.3), ASF and NRR for different uniform content popularity distributions, under: (**a**) LCE caching policy, (**b**) *Betw* caching policy and (**c**) *ProbCache* caching policy. Content popularity is modelled according to the Mandelbrot-Zipf (MZipf) distribution with the settings: p=5.0,α=0.0,0.3,0.6,0.91.2. Each CR is equipped with a cache size of 0.4% of the content catalogue. The results reported are the average of 20 randomized simulation runs.

In Figure 6 and Figure 7, we compare the three forwarding strategies and show the impact of the content popularity model. The request distribution is uniform in the network, and we consider 5 different values of the skew parameter (α=0.0,0.3,0.6,0.9,1.2), including α=0 which corresponds to equally popular items. The closer the skew parameter is to 0, the more ineffective in-network caching will be for the same catalogue size. Our observations confirm this because as most of the requests concentrate on a smaller proportion of the content catalogue, that is, as α increases, all the forwarding strategies can hit more local caches. Also for α=0.0 and α=0.3, both ASF and NDNFS-RLRNN deliver almost identical performance with respect to the cache hit rate under the different caching policies. In fact, under the *Betw* policy, ASF is slightly better than NDNFS-RLRNN for these values of α in terms of the cache hit rate. However, as α is increased from 0.6 to 1.2, NDNFS-RLRNN produces a performance improvement of between 27–43% compared with ASF.

Finally, our results suggest that for ubiquitous caching policies like LCE and *ProbCache*, increasing the exploration in our proposed forwarding strategy, can deliver improved results with respect to exploiting in-network caching, at the cost of higher overhead as shown by the effect of increasing the probing parameter in Figure 3. While the above statement is also true for the *Betw* policy, interestingly, it is only under this policy that the NDNFS-RLRNN performs better than the NRR despite using significantly lower probing. Since *Betw* mostly caches in specific nodes in the network, this demonstrates NDNFS-RLRNN’s intelligence to search in the most worthwhile areas more efficiently than an approach that searches the entire network.

## 7. Conclusions and Further Work

This paper has addressed Information Centric Networks in the framework of Named Data Networking (NDN). We have proposed an adaptive forwarding strategy, NDNFS-RLRNN for the NDN architecture which employs an online learning algorithm, reinforcement learning using the random neural network, to forward Interest packets. Our proposed approach is dynamic and does not persist on the links put forward by the routing protocol, so that it may better recognize the role of the forwarding plane to take advantage of the in-network caching capability of the NDN architecture.

In our tests, we compare NDNFS-RLRNN with two other forwarding strategies: one that restricts forwarding and probing of Interests to the interfaces suggested by the routing protocol and another that broadcasts each Interest. We also considered three different caching policies from the literature to evaluate the forwarding strategies under different caching behaviours. We consider two ubiquitous caching policies exhibiting different cache eviction rate properties and a policy that bases its caching decisions on the network topological information.

Our results suggest that when in-network caching is effective, NDNFS-RLRNN strategy can achieve better delivery than a forwarding strategy that persists on the existing static routing layer preferences and a more efficient performance than a nearest replica forwarding strategy that floods requests. Furthermore, the results also indicate that for ubiquitous caching policies, NDNFS-RLRNN’s performance can be significantly improved by increasing its exploration of the network. For a policy that caches contents at specific nodes based on the network topology, we see that the rate of improvement in terms of exploiting in-network caching reduces as the probing frequency of NDNFS-RLRNN increases. However, the results also show that even with a low probing frequency, NDNFS-RLRNN produces the best performance compared with the other forwarding strategies under this policy.

Future work will focus on evaluating our approach for congested systems, and also in the presence of denial of service attacks. Such evaluations can also lead to improvements in the adaptive on-line policy and result in a better evaluation of the computational overhead of our adaptive on-line approach.

## Figures and Tables

**Table 1:** Notation for the RNN model.

Notation	Definition
k(t)	State vector of the RNN at time *t* where $k_{i}\left(t\right)\geq 0$ is the potential level of a neuron *i* in the network
$r_{i}$	Firing rate at neuron *i*
$\Lambda_{i}$	Arrival rate of positive exogenous signals at neuron *i*
$\lambda_{i}$	Arrival rate of negative exogenous signals at neuron *i*
$p^{+}\left(i,j\right)$	Probability that a signal leaving neuron *i* is excitatory and heads for neuron *j*
$p^{-}\left(i,j\right)$	Probability that a signal leaving neuron *i* is inhibitory and heads for neuron *j*
d(i)	Probability that a signal leaving neuron *i* departs the network
